# Germline Stem Cell Gene *PIWIL2* Mediates DNA Repair through Relaxation of Chromatin

**DOI:** 10.1371/journal.pone.0027154

**Published:** 2011-11-16

**Authors:** De-Tao Yin, Qien Wang, Li Chen, Meng-Yao Liu, Chunhua Han, Qingtao Yan, Rulong Shen, Gang He, Wenrui Duan, Jian-Jian Li, Altaf Wani, Jian-Xin Gao

**Affiliations:** 1 Laboratory of Tumorigenesis and Immunity, Clinical Stem Cell Research Center, Renji Hospital, Shanghai Jiao Tong University School of Medicine, Shanghai, China; 2 Department of Pathology, Ohio State University Medical Center, Columbus, Ohio, United States of America; 3 Department of Radiology, Ohio State University Medical Center, Columbus, Ohio, United States of America; 4 Comprehensive Cancer Center, Ohio State University Medical Center, Columbus, Ohio, United States of America; 5 Department of General Surgery, the First Affiliated Hospital of Zhengzhou University, Zhengzhou, Henan, China; 6 Department of Radiation Oncology, University of California Davis, Sacramento, California, United States of America; Brunel University, United Kingdom

## Abstract

DNA damage response (DDR) is an intrinsic barrier of cell to tumorigenesis initiated by genotoxic agents. However, the mechanisms underlying the DDR are not completely understood despite of extensive investigation. Recently, we have reported that ectopic expression of germline stem cell gene *PIWIL2* is associated with tumor stem cell development, although the underlying mechanisms are largely unknown. Here we show that *PIWIL2* is required for the repair of DNA-damage induced by various types of genotoxic agents. Upon ultraviolet (UV) irradiation, silenced *PIWIL2* gene in normal human fibroblasts was transiently activated after treatment with UV light. This activation was associated with DNA repair, because *Piwil2*-deficienct mouse embryonic fibroblasts (mili^-/-^ MEFs) were defective in cyclobutane *pyrimidine dimers* (CPD) repair after UV treatment. As a result, the UV-treated mili^-/-^ MEFs were more susceptible to apoptosis, as characterized by increased levels of DNA damage-associated apoptotic proteins, such as active caspase-3, cleaved Poly (ADP-ribose) polymerase (PARP) and Bik. The impaired DNA repair in the mili^-/-^ MEFs was associated with the reductions of histone H3 acetylation and chromatin relaxation, although the DDR pathway downstream chromatin relaxation appeared not to be directly affected by Piwil2. Moreover, guanine–guanine (Pt-[GG]) and double strand break (DSB) repair were also defective in the mili^-/-^ MEFs treated by genotoxic chemicals Cisplatin and ionizing radiation (IR), respectively. The results indicate that Piwil2 can mediate DNA repair through an axis of Piwil2 → histone acetylation → chromatin relaxation upstream DDR pathways. The findings reveal a new role for Piwil2 in DNA repair and suggest that Piwil2 may act as a gatekeeper against DNA damage-mediated tumorigenesis.

## Introduction

*PIWIL2* (*Piwi-like 2)* gene (alias *mili* in mouse or *hili* in humans), a member of AGO/PIWI gene family, is exclusively expressed in the germline stem cell (GSC) of testis but not in the adult tissue stem cells and somatic cells [1], [2], [3], [4]. Recently, expression of PIWIL2 has been widely detected in a variety of tumor cell lines as well as in various stages of primary cancers [5], [6], [7], [8], [9], [10], [11]. Interestingly, *PIWIL2* gene can be alternatively activated in tumor cells by intragenic promoters, resulting in a number of Piwil2 variants, namely Piwil2-like (PL2L) proteins with a potential function in tumorigenesis [11]. Especially, we have found that PIWIL2 expression is associated with the development of tumor stem cell (TSCs) [6], [11], [12], [13], [14]. However, the exact mechanisms *PIWIL2*-mediated cell transformation and tumor formation is unknown.

The AGO/PIWI family proteins containing PIWI and PAZ domains (PPD) [1], [2] show multiple biological functions. Although it is known that the PAZ domain is bound by siRNA [15], the function of PIWI domain has not been clarified [16]. The Piwil2 protein is shown to be essential for gametogenesis in various organisms [3]. It controls gametogenesis through regulating self-renewal [17], RNA silencing [18], [19], translational regulation [4], chromatin remodeling [20], [21] and epigenetic modifications of GSCs [21], [22]. Piwil2 binds piwi-interacting RNA (piRNA) to silence the selfish genetic elements such as retrotransposons through methylation of cytosine of CpG islands in the germ cells of testis [22], [23], [24]. Dysregulated or ectopic expression of Piwi family proteins, especially Piwil2, seems linked to cell transformation and tumorigenesis [6], [11], [12], [13]. Elucidation of the role of Piwil2 in signaling cell transformation and tumorigenesis will provide new insights into the biological functions of *PIWIL2* and potential therapeutic targets in cancer treatment.

Genotoxic agents-induced DNA damage is a primary cause of tumorigenesis [25], [26]. The resulted DNA damage response (DDR) is an anti-cancer barrier in early human tumorigenesis [26]. However, the cell-intrinsic mechanisms that serve as a barrier to tumorigenesis during tumor development are still not completely understood despite of the extensive investigations on cancer genes last decades. DDR is a coordinated process between the events of biochemical pathways for DNA repair, chromatin remodeling, cell cycle arrest and/or apoptosis [27], [28], [29]. Different types of DNA damage, including DNA modification or base damage, crossing linking and single- and double-strand breaks (SSBs and DSBs), can be induced by ionizing radiation (IR), ultraviolet (UV) light, chemotherapeutic agents and even aberrant chromatin remodeling [30]. IR is a more clinically relevant to DNA DSB inducer. Continuous formation of DNA DSBs may contribute to the genomic instability that characterizes the vast majority of human cancers [31]. The efficacy of DNA repair in mammalian cells is vital for the genomic integrity and genomic functions, a collection of processes by which a cell identifies and corrects damages to DNA molecules and prevents against oncogenetic mutations and potential cell trasnformation [27], [28]. Chromatin relaxation and remodeling are critical for the initiation of DNA repair [32], [33]. Failure to repair damaged DNA may incur senescence, apoptosis (cell suicide), and deregulated cell division that leads to cell transformation and tumor formation [25], [26], [34].

In this study, we demonstrate that Piwil2 can be activated upon DNA damage and is required for DNA repair following DNA damages induced by IR, UV light, and cisplatin. The Piwil2-mediated DNA repair appears to be associated with histone H3 acetylation that is required for chromatin relaxation, a critical and initial step for DNA repair. The results demonstrated a new role of Piwil2 in mammalian cells for DNA repair and provide the evidence of Piwil2 as the rate-limiting with cell-intrinsic barrier to tumorigenesis.

## Results

### *PIWIL2* gene is activated upon DNA damages

**Figure 1:**
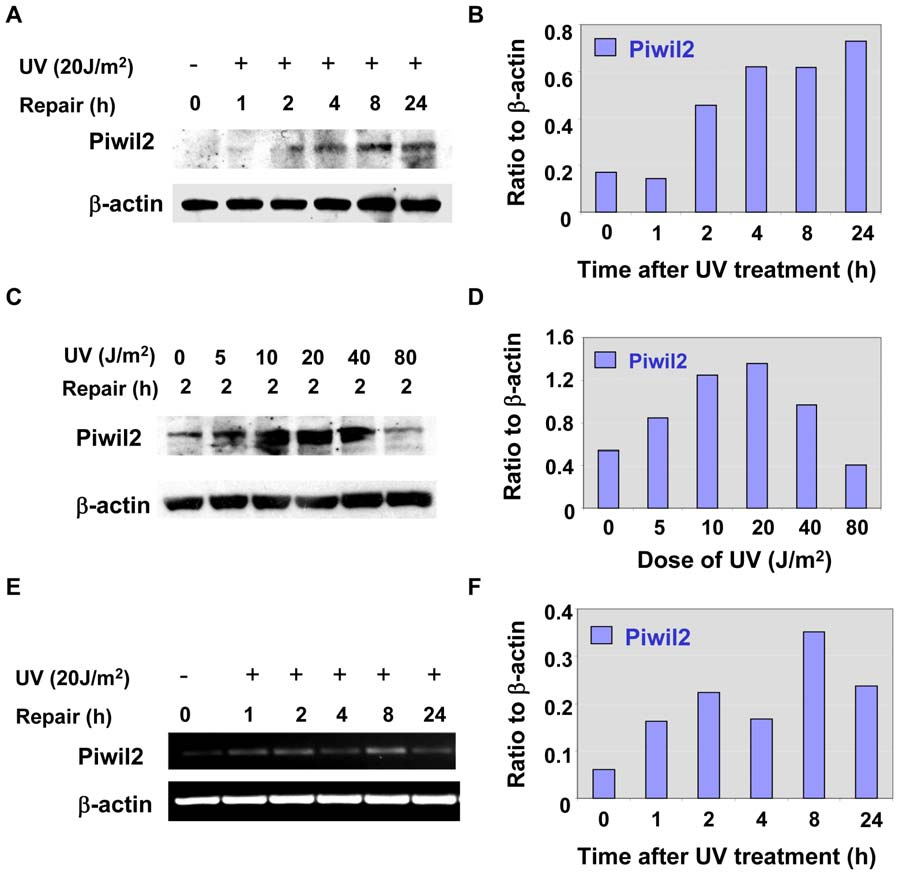
UV irradiation induces Piwil2 expression in HDFs. **A &B.** Kinetics of Piwil2 expression in responding to DNA damage induced by UV light. HDFs were irradiated with UV (20 J/m^2^) and harvested at 0, 1, 2, 4, 8 and 24 h later and analyzed by Western blotting for piwil2 expression, using polyclonal rabbit antibody to Piwil2 (1∶1000 dilution). **C & D.** Dose-dependent expression of Piwil2 in responding to UV-induced DNA damage. HDFs were treated with various dose of UV, harvested 2 hrs after treatment and analyzed by Western blotting for Piwil2 expression. **E & F.** HDFs were treated as in A, and analyzed by RT-PCR for Piwil2 transcript expression. A, C & E: micrographs of Piwil2 proteins or transcripts; B, D & F: quantitation of the Piwil2 proteins or transcripts in A, C & E by normalization to β-actin. The data shown are a representative of two experiments.

To determine the response of *PIWIL2* gene to DNA damages, we treated human dermal fibroblasts (HDFs) with various doses of UV light, and examined the expressions of Piwil2 transcripts and proteins in these cells at various time points by Western-blotting and RT-PCR. As shown in Figure 1, PIWIL2 protein expression in human dermal fibroblasts (HDFs) was induced by UV irradiation as early as one hour after treatment (Fig. 1A–B). The expression was dose-dependent and reached a peak between 10–20 J/m^2^ UV irradiation 2 hrs after treatment (Fig. 1C–D). However, PIWIL2 expression was individually variable with experiments being at the high dose of 80 J/m^2^ and sometime the level of PIWIL2 was lower than at 40 J/m^2^, probably associated with more cell death at this time point (Fig. 1C and not shown). Consistently, Piwil2 transcripts were also up-regulated in HDFs as early as one hour after UV treatment (Fig. 1E–F). Interestingly, the level was temporarily reduced at 4 hrs, then reached a peak at 8 hrs after treatment and decreased thereafter (Fig. 1E–F). After 48–72 hrs of treatment, Piwil2 transcripts go back to the baseline, regardless of the level of PIWIL2 proteins (not shown). The results suggest that *PIWIL2* gene can be activated temporarily upon DNA damages, and Piwil2 expression is transcriptionally regulated.

### Piwil2-deficiency promotes DNA damage-induced cell death

**Figure 2:**
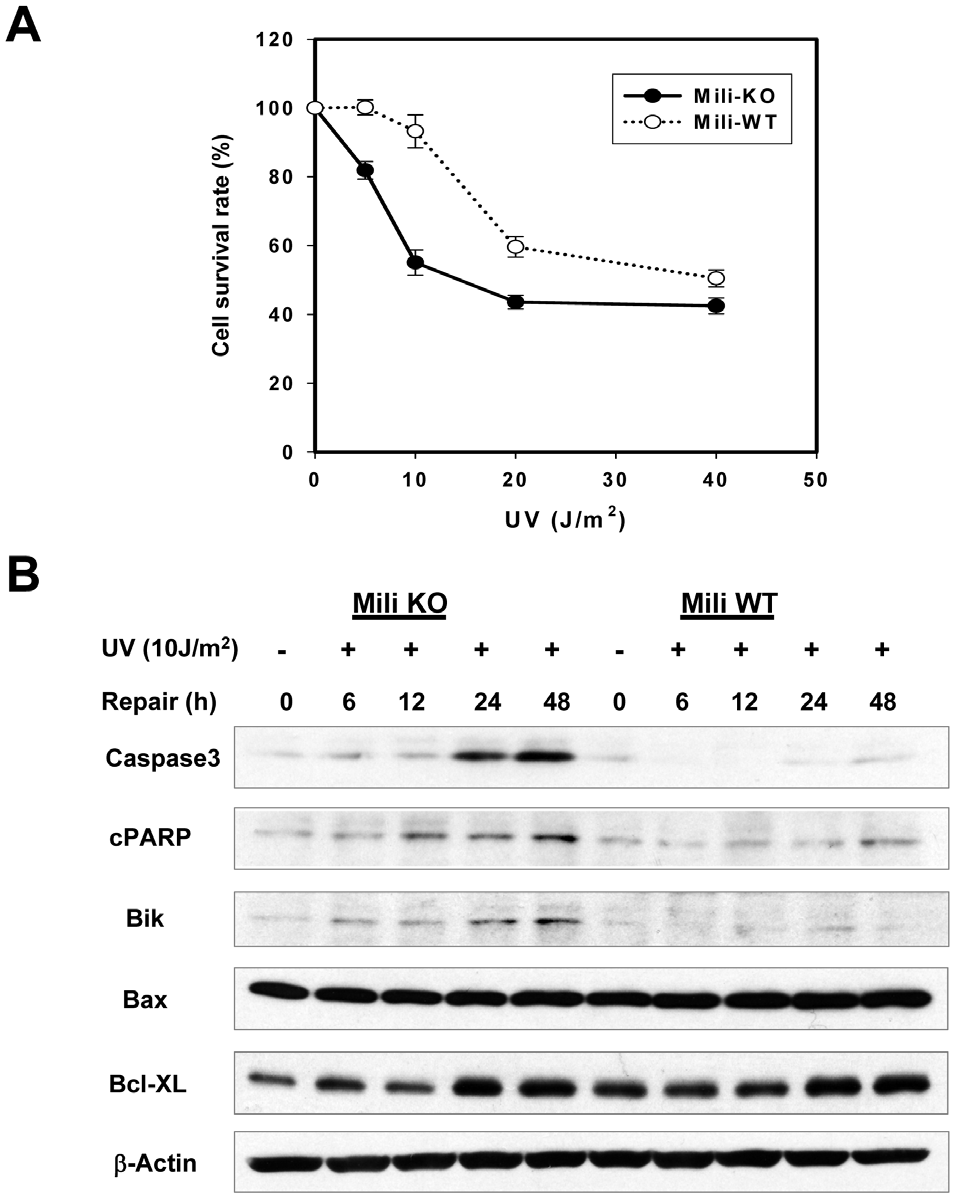
Mili^-/-^ MEFs are more susceptible than WT MEFs to apoptosis induced by UV light. **A.** Survival rate was significantly decreased in the mili^-/-^ MEFs treated with various doses of UV (p<0.01). The relative cell survival rate was determined by methylene blue staining 72 or 96 hours after treatment. Shown are the data derived from 96 hrs after irradiation. **B**, DNA damage-associated apoptotic proteins were up-regulated in the UV-treated mili^-/-^ MEFs. The data shown are a representative of three experiments in triplicate. Caspase-3: activated caspase-3; cPARP: cleaved PARP.

To determine the significance of Piwil2 responding to DNA damage, we investigated effects of Piwil2 on DNA damage-induced cell death, using mouse embryonic fibroblasts (MEFs) derived from mili knockout (KO) mice. As observed in HDFs, Piwil2 expression was also also up-regulated in MEFs upon UV irradiation (data not shown). To determine the susceptibility of mili^-/-^ MEF to apoptosis induced by UV light, we evaluated cell survival rate after UV treatment. As shown in Fig. 2, the survival rate at day 4 of mili^-/-^ MEFs were significantly reduced in responding to various doses of UV light, compared to that of wild-type (WT) MEFs. This was associated with increased apoptosis of the UV-treated mili^-/-^ MEF, because DNA damage-associated apoptotic proteins including activated caspase-3, cleaved Poly (ADP-ribose) polymerase (PARP) and Bik were up-regulated in the mili^-/-^ MEFs; however, the expression of Bax and Bcl-XL, which are not specifically associated with DNA damage, was not significantly different between mili^-/-^ and WT MEFs (Fig. 2B). Especially the up-regulation prominently occurred after 12 h of UV treatment when damaged DNA should have been repaired, suggesting that DNA repair might have failed in the mili^-/-^ MEFs.

### Piwil2 is essential for DNA repair

**Figure 3:**
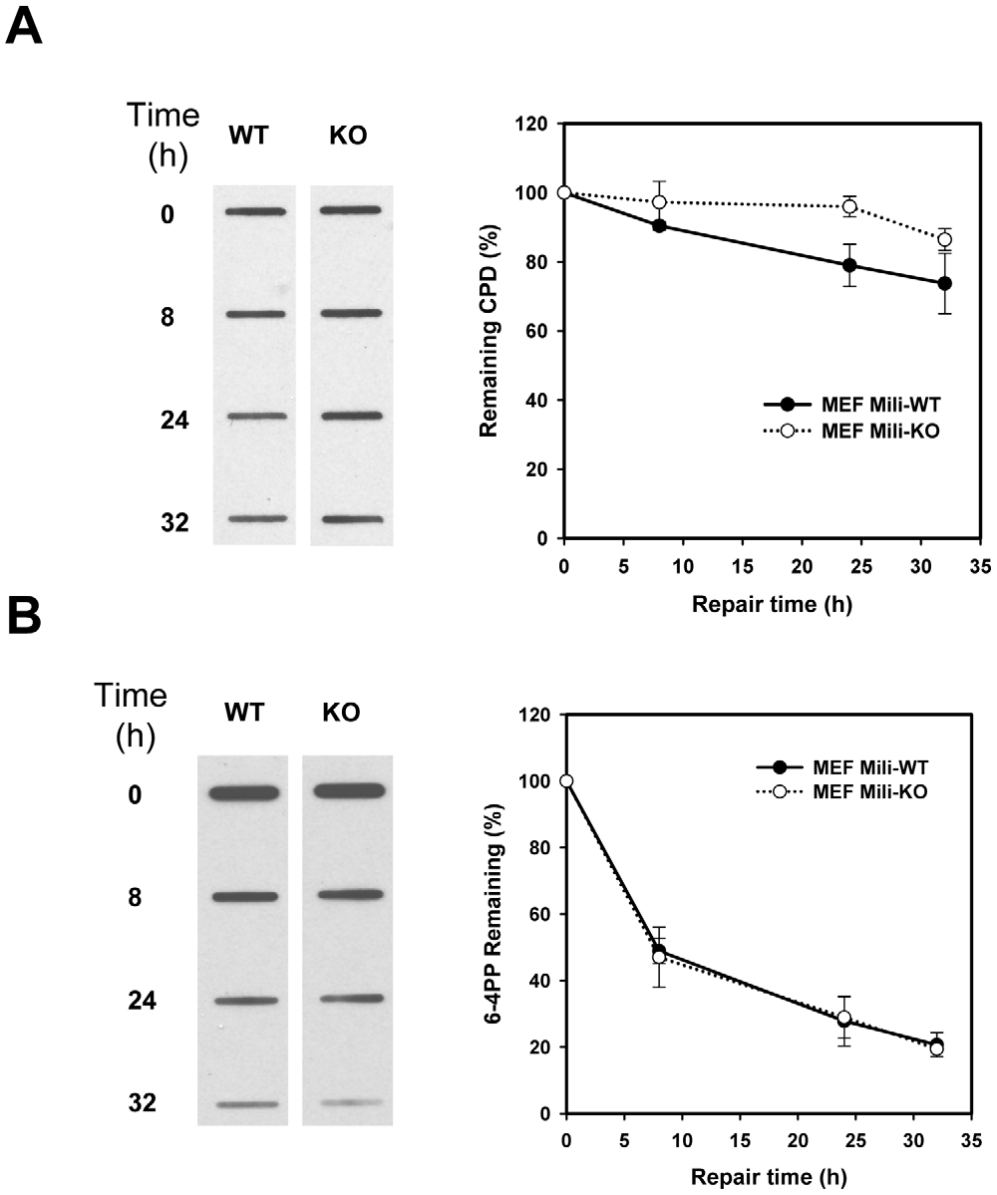
Piwil2 is required for repair of DNA damage induced by UV light. Mili^-/-^ (KO) and WT MEFs were treated with 10 J/m^2^ UV light and examined for CPD (**A**) and 6–4 PP abducts (**B**) at various time points, using Immuno-slot blotting. The data shown are a representative of two experiments. **, p<0.01.

To verify that DNA repair was defective in the UV-treated mili^-/-^ MEF, we treated mili^-/-^ and WT MEFs with UV light, and examined cyclobutane *pyrimidine dimers* (CPD) and 6–4 pyrimidine photoproducts (6–4 PP), which can be induced by UV irradiation through covalent-linkage between adjacent cytosine and thymine bases [35], [36]. However, 6–4 PP is only 10–15% of the damaged DNA induced by UV light [37]. As shown in Figure 3, CPD repair was significantly reduced in mili^-/-^ MEFs, compared to that in WT MEFs during DNA repair (Fig. 3A). Interestingly, 6–4 PP in mili^-/-^ MEFs was reduced to the same level as observed in WT MEFs (Fig. 3B). Despite of this, the results suggest that Piwil2 activation upon DNA damage is responsible for DNA repair. Lack of Piwil2 may lead to defective DNA repair, resulting in decreased cell survival rate because of increased apoptosis (Fig. 2A).

### Piwil2 mediates chromatin relaxation through regulating histone acetylation

**Figure 4:**
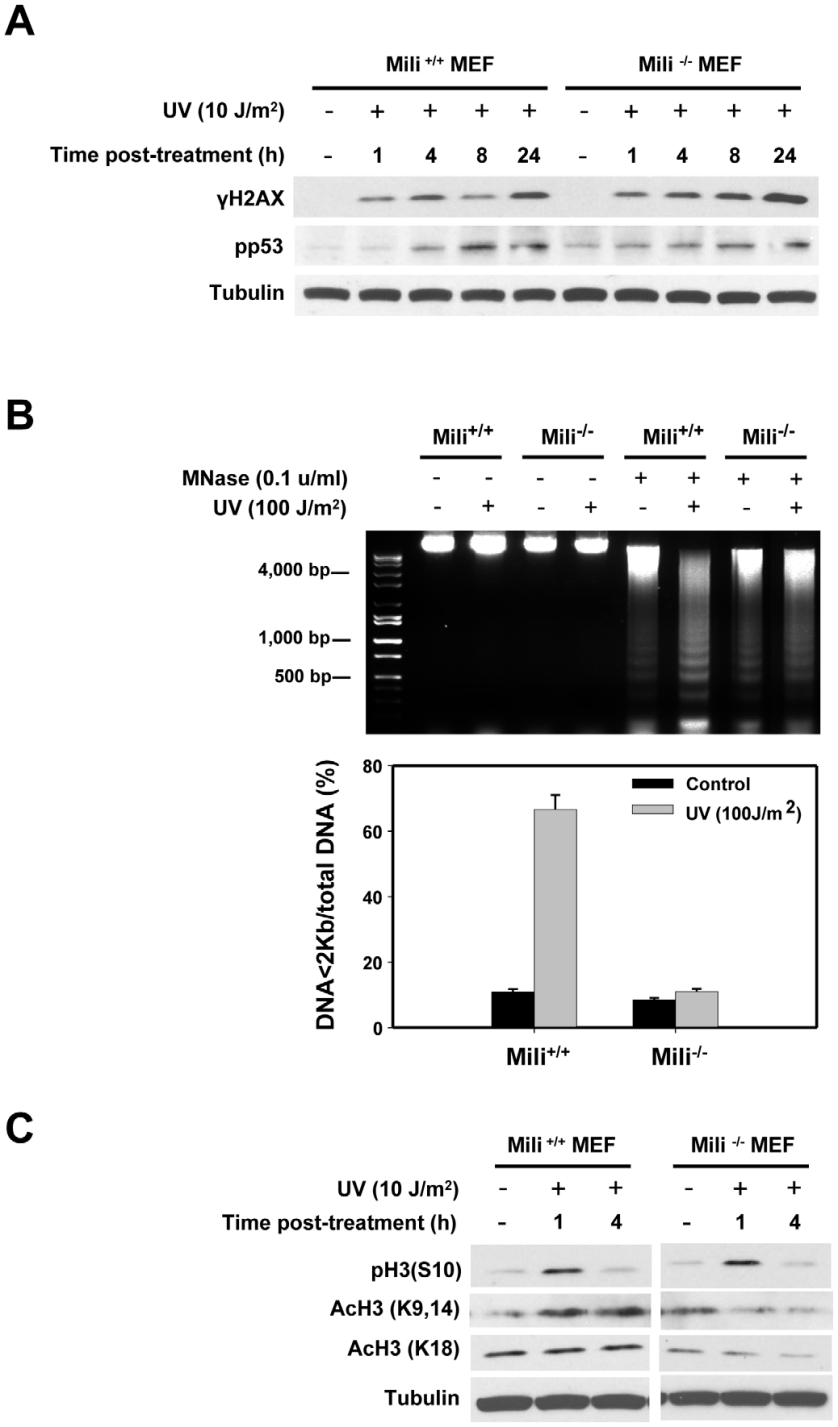
Piwil2 promotes chromatin relaxation through regulation of histone H3 acetylation in responding to DNA damage. **A.** Piwil2 has no effect on activation of H2AX and p53 in MEFs after treatment with UV light. The γH2AX and pp53 in mili^-/-^ and WT MEFs were analyzed by Western blotting. **B**. Piwil2 is required for chromatin relaxation in MEFs irradiated by UV light, as revealed by MNase assay. Top panel: micrograph of DNA ladders; bottom panel: quantitation of DNA fragments in the top panel. **, p<0.01. **C.** Piwil2 up-regulate histone H3 acetylation in MEFs irradiated by UV light. Expression of phosphorylated histone H3 [pH3 (S10)] and acetylated histone H3 [AcH3 (K9, 14) and AcH3k18] in mili^-/-^ and WT MEFs were analyzed by Western blotting after UV irradiation. Tubulin expression was monitored as an internal control. Shown are the data from one of two experiments.

The impaired DNA repair in mili^-/-^ MEFs might be associated with abnormal DDR. To determine the mechanisms underlying Piwil2-mediated DNA repair, we examined whether DDR signal transduction pathways were affected by Piwil2. H2AX and p53 are two hallmarks of DDR signal transduction pathways [38], [39], which are usually phosphorylated for DNA repair during DDR. Unexpectedly, phosphorylated H2AX (γH2AX) and p53 (pp53) were not significantly reduced in mili^-/-^ MEFs after UV irradiation (Fig. 4A), suggesting that DDR signal transduction pathways are unlikely affected by Piwil2 deficiency. This appeared to be true, because the phosphorylation of both H2AX and p53 was neither affected in the mili^-/-^ MEFs after treatment with cisplatin, a genotoxic agent used for cancer chemotherapy [40] (data not shown).

An immediate change of DDR is chromatin relaxation, which promotes accessibility of DDR proteins to the lesions of DNA [41]. Since Piwi proteins is associated with chromatin remodeling in various organisms [21], [42], we hypothesized that Piwil2 might involve in chromatin remodeling upon DNA damage. Thus, we examined the state of chromatin condensation in mili^-/-^ MEFs upon DNA damage. Chromatin condensation was evaluated by digestion with micrococcal nuclease (MNase), which preferentially cuts the DNA in the linker regions between nucleosomes, releasing chromatin fragments containing different numbers of nucleosomes [32], [43]. In mili^-/-^ MEFs, chromatin accessibility to MNase was blocked, because the chromatin in the UV-treated mili^-/-^ MEFs was not digested by MNase, demonstrating a compact DNA ladder in agrose gel and contrasting to that in WT MEFs (Fig. 4B). The results suggest that Piwil2 is required for transforming condensed chromatin into a more relaxed structure, which is associated with active gene transcription [44].

It has been suggested that histone H3 acetylation is required for chromatin relaxation [41], [44]. Thus, we hypothesized that histone acetylation might be inhibited in the DNA-damaged mili^-/-^ MEFs. To verify the hypothesis, we examined the status of histone H3 acetylation in mili^-/-^ MEFs. As expected, the acetylation of H3K9, 14 (acH3K9/14) and acH3K18 was reduced in mili^-/-^ MEFs after UV treatment, while acH3K9/14 was increased in WT MEFs (Fig. 4C). It should be noted that mili^-/-^ MEFs expressed higher level of acH3K9/14 than WT MEFs before UV treatment (Fig. 4C). The results confirm that decreased chromatin relaxation in mili^-/-^ MEFs is associated with reduced acetylation of histone H3. However, Piwil2 had no effect on histone H3 phosphorylation, because the level of pH3(S10) was not significantly changed in mili^-/-^ MEFs compared to that in WT MEFs (Fig. 4C).

### Piwil2-mediated DNA repair is of broad significance

**Figure 5:**
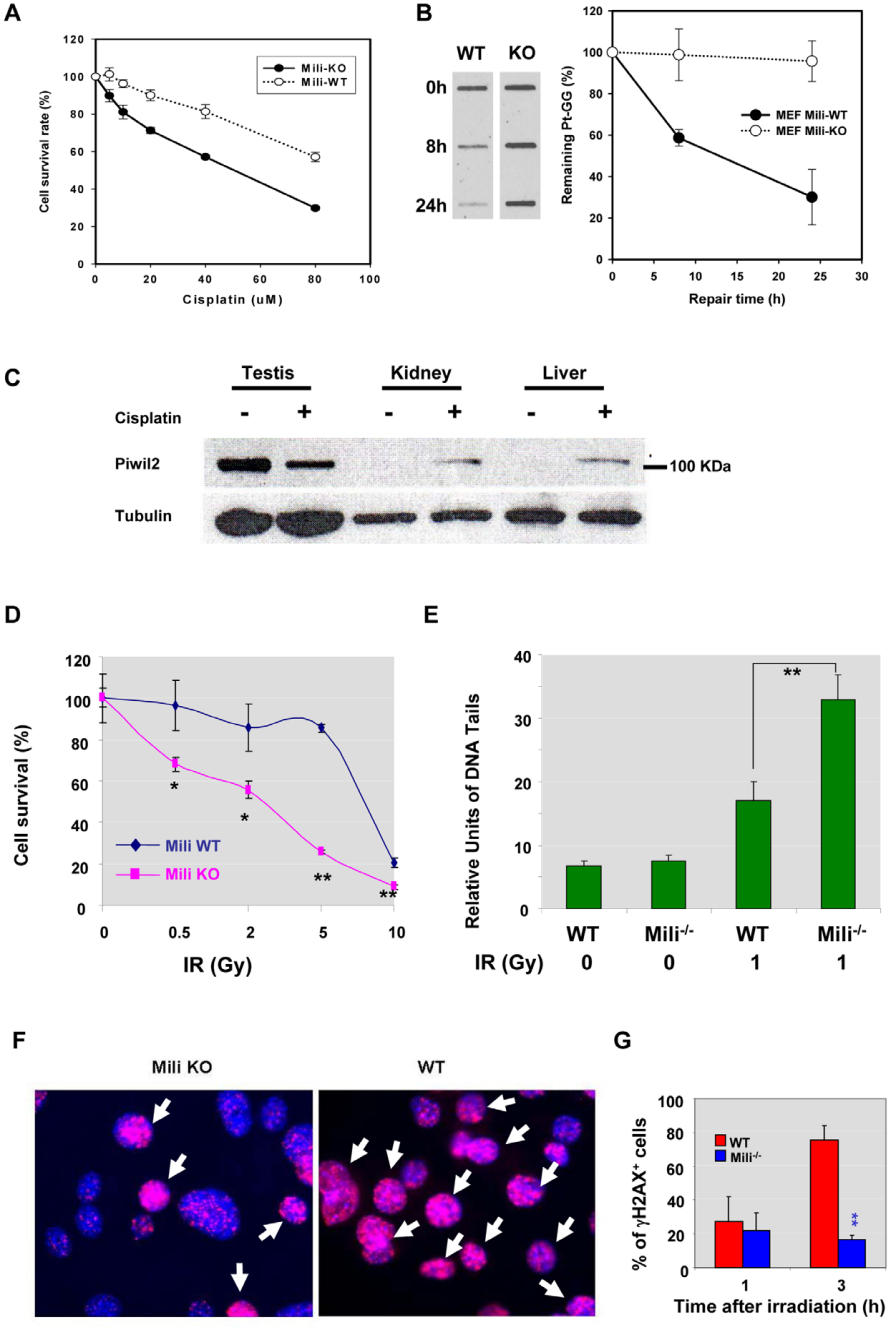
Piwil2 is required for repair of DNA damage induced by IR and cisplatin. **A, B & C.** Piwil2 is required for repair of DNA damage induced by cisplatin. (**A**) The survival rate of mili^-/-^ MEFs was significantly reduced in a dose-dependent manner as compared to WT MEFs after cisplatin treatment in various doses. The relative cell survival rate was determined by methylene blue staining (n = 3). **, p<0.01. (**B**) DNA repair in the cisplatin-treated mili^-/-^ and WT MEFs. The MEFs were treated with cisplatin for 1 h, cultured and harvested at the indicated time for ISB assay to determine amounts of Pt-GG in the cells (n = 3). (**C**) Cisplatin induced Piwil2 expression *in vivo*. Male mice were treated i.p. with cisplatin (20 mg/m^2^) or vehicle (PBS) for 5 consecutive days and kidney, liver and testis were harvested and whole cell lysates from the tissue were prepared and subjected to Western blotting with monoclonal anti-piwil2 IgM antibody (Kao2 supernatant; 1∶50). The data shown were a representative of two experiments. **D & E.** Piwil2 is required for repair of DNA damage induced by IR. (**E**) Mili^-/-^ and WT MEFs were seeded at 1×10^5^/well in 6-well plates in triplicates. When cells grew to 50–60% confluence (2 days) they were exposed to various doses (0, 0.5, 2, 5, 10 Gy) of X-ray (RS 2000 Biological Irradiator; Rad Source Technologies, Inc. Alpharetta, GA). Four days after irradiation, cells were harvested and counted with trypan blue exclusion of dead cells. Cell survival rate was calculated as percentage of viable cells of each dose normalized to untreated counterparts (n = 3). *, p<0.05; **, p<0.01. (**E**) DNA repair in IR-treated MEFs. Mili^-/-^ and WT MEFs were X-rayed at exponential growth phase and comet assay was performed with standard protocol. DNA damage was estimated by measuring the distance of the tail against the edge of far side of the nuclei for 50 random selected cells (n = 50; **, p<0.001). The data shown are representative of two experiments. **F & G.** Different size of γH2AX foci in Mili^-/-^ MEFs versus WT MEFs irradiated by X-ray. (**F**) Representative micrographs of γH2AX foci in MEFs at 3 h after X-ray irradiation (3 Gy). Arrows indicate the MEFs with large γH2AX foci; (**G**) Quantitation of γH2AX foci in MEFs at 1 and 3 h after X-ray irradiation (n = 3). **, p<0.01 compared between Mili^-/-^ and WT MEFs. Note that there is no significant difference between Mili^-/-^ and WT MEFs in the formation of large γH2AX foci at 1 h after irradiation.

To determine whether the Piwil2-meidated DNA repair is universal to DNA damage induced by different genotoxic agents, we investigate the DNA repair in mili^-/-^ MEFs treated by cisplatin and ionizing radiation (IR), respectively. As shown in Fig. 5A, cell survival rate of mili^-/-^ MEFs were significantly reduced compared to WT counterparts after treatment with various doses of cisplatin. Cisplatin can cause intrastrand crosslinking of DNA to form abducts such as guanine–guanine (Pt-[GG]), which can be detected by mAbs [45]. Consistently, the level of Pt-[GG] was not significantly reduced in the cisplatin-treated mili^-/-^ MEFs at 8 and 24 hrs of treatment, as compared to the level of Pt-[GG] in the cisplatin-treated WT MEFs (Fig. 5B). The Piwil2-responding to cisplatin was also observed *in vivo* (Fig. 5C). Piwil2 was detected in the kidney and liver of mice treated with cisplatin but not with vehicle (Fig. 5C). The results suggest that *PIWIL2* can respond to cisplatin-induced DMA damage.

Similar results were also observed in the mili^-/-^ MEFs treated by X-ray radiation or IR, which can induce DNA DSBs (Fig. 5D–E). The cell survival rate of X-ray-treated mili^-/-^ MEFs was significantly decreased in a dose-dependent manner, as compared to their WT counterparts (Fig. 5C). The reduced survival rate appeared to be associated with their reduced capacity of DNA repair, as revealed by Comet assay (Fig. 5E). Moreover, consistently with the observation that phosphorylation of H2AX was not affected in the mili^-/-^ MEFs treated by UV and cisplatin, phosphorylation of H2AX was neither affected in the X-ray treated mili^-/-^ MEFs, because the size of γH2AX foci was comparable between the mili^-/-^ MEFs and WT MEFs at 1 hour after the treatment (Fig. 5G). However, the size of γH2AX foci in the majority of mili^-/-^ MEFs was much smaller than that in the WT MEFs at 3 hrs of X-ray treatment (Fig. 5F–G), suggesting that Piwil2 did not affect phosphorylation of H2AX, but did affect the formation of chromatin remodeling complexes [46], which mediate DNA DSB repair [29]. Taken together, these results confirm that Piwil2 is essential for DNA repair in the cells insulted by various types of genotoxic agents, including UV, IR, and chemotherapeutic agents such as cisplatin.

## Discussion

Normally, *PIWIL2* gene is silent in adult tissue stem cells and somatic cells except for testis [1], [4], [5], [11]. Recently we and others have found that Piwil2 may play important roles in tumor development, despite the fact that the underlying mechanisms are not yet clear [5], [7], [8], [9], [10], [11], [13], [14]. In this study, we have for the first time revealed that *PIWIL2* gene can be activated upon DNA damages induced by genotoxic agents. The finding suggests that the usually silent *PIWIL2* gene in adult tissue cells is responsible for cell stresses and thus can be activated upon DNA damage. The notion is further supported by our observation that variable levels of Piwil2 transcripts and proteins were sometimes detected in HDFs and other cell lines in the long-term cultures, probably associated with increased stressing in the cultures such as high density or over growth of cells (not shown). This activation is critical for DNA repair, because DNA repair was defective in the mili^-/-^ MEFs treated by various types of genotoxic agents, including UV, IR and cisplatin. Consistently with the failure to repair damaged DNA, increased apoptosis or decreased cell survival was observed in mili^-/-^ MEFs treated by these agents. Interestingly, activated caspase-3, cleaved PARP and Bik but not Bax were up-regulated in mili^-/-^ MEFs after UV treatment, suggesting that the DNA damage-associated apoptotic pathway is activated preferentially [47], [48], [49]. Therefore, Piwil2 is required for DNA repair.

**Figure 6:**
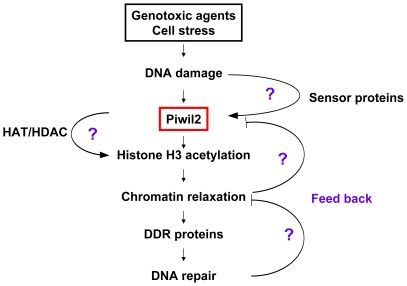
Schematic diagram of the role of Piwil2 for DNA repair. Once DNA damage is induced by genotoxic agents, silent *PIWIL2* gene is activated, modulating chromatin relaxation through histone H3 acetylation to allow DNA damage signaling proteins and DNA repair proteins migrate to the sites of DNA damage. Thus, Piwil2 might control multiple down-stream pathways for DNA repair. It is possible that *PIWIL2* could be activated by DNA damage sensor proteins to regulate histone H3 acetylation through effecting on HAT and/or HDAC. In addition, the proteins recruited to DNA damage sites might in turn suppress chromatin relaxation and Piwil2 expression after success of DNA repair (negative feedback). Overall, Piwil2 may mediate DNA repair through an axis of Piwil2 → histone acetylation → chromatin relaxation up-stream of DDR. DDR: DNA damage response; HAT: histone acetyltransferases; HDAC: histone deacetylase.

Genotoxic agents-induced DNA damage is immediately followed by complex DDR cascades, including two major events: chromatin relaxation and the recruitment of DDR proteins, i.e., DNA damage signaling proteins and DNA repair proteins, to the sites of DNA damage [28], [32], [41], [46]. Chromatin relaxation allows the DDR proteins to be recruited to the site of DNA damage and thus is a prerequisite for DAN repair [41]. There are multiple pathways for DNA-damage repair, including direct reversal (DR), base-excision repair (BER), nucleotide excision repair (NER) and DNA mismatch repair (MMR) for single-strand break (SSB), and homologous recombination (HR) and non-homologous end joining (NHEJ) for double-strand break (DSB) repair [50], [51] (Fig. 6). In this study, we demonstrated that DNA repair in mili^-/-^ MEFs was defective and this defect is associated with compact structure of chromatin but not with activation of signaling transduction proteins for DNA damage. Piwil2 modulates chromatin relaxation through promoting histone H3 acetylation during DDR, because acH3K9/14 and acH3K18 were reduced in mili^-/-^ MEFs after DNA damage. It is well known that histone acetylation is associated with transcriptional activation and euchromatin formation or chromatin relaxation [29], [44]. The unwound heterochromatin allows the damaged DNA to be accessible for the signaling transduction proteins of DNA damage as well as DNA repair proteins [25], [26], [52]. The finding is consistent with the functions of Piwi proteins to promote chromatin remodeling in *Drosophila* [20], [53]. It is unlikely that Piwil2 is directly involved in the activation of DDR proteins, because we did not observe any effect of Piwil2 on the activation of p53 and H2AX, two hallmarks for the signaling transduction pathway of DNA damage. However, the size of γH2AX foci in the IR-treated mili^-/-^ MEFs was greatly reduced compared to that in the WT counterparts, suggesting that the formation of chromatin remodeling complexes was defective in mili^-/-^ MEFs during DSB repair. This may be caused by defective chromatin decondensation in mili^-/-^ MEFs, which limited the recruitment of γH2AX and then DNA repair proteins to the intra-strand sites of DSB, resulting in small γH2AX foci. The mechanisms underlying the phenomenon need further investigation. Here, we propose that Piwil2 mediates DNA repair through promoting chromatin relaxation during DDR (Fig. 6).

In addition to histone H3 acetylation, the mechanisms underlying Piwil2-mediated chromatin relaxation need further investigation. Chromatin relaxation may not only allow the access of DDR proteins to DNA damage sites but also the transcription of genes required for DNA repair. In addition, it has recently been reported that Piwil2 can regulate translation in germline stem cells to maintain their self-renewal [4]. This might also happen in DNA damaged cells. It is interesting to elucidate whether the decreased level of acetylated histone H3 in the DNA damaged mili^-/-^ MEFs is associated with increased activity of histone deacetylases (HDACs) or decreased histone acetyltransferases (HATs) and how, if any, Piwil2 regulates the activity of HDACs and HATs upon DNA damage. Many factors that are associated with HAT or HDAC activity and DDR proteins have been reported to modulate chromatin relaxation during DNA repair, such as ATM (ataxia telangiectasia, mutated), high mobility group 1 Protein, NG1b, and TIP60 [32], [41], [54], [55], [56]. These factors might be the clues for elucidating how Piwil2 regulates HAT and/or HDAC activity.

Various types of DNA damage, including DNA crossing linking, SSB, DSB, and replication errors, can be induced by different genotoxic agents, such as UV light, IR, chemotherapeutic agents and endogenous cellular metabolism [28], [29], [57]. UV light mainly causes cross-linking between adjacent cytosine and thymine bases, producing cyclobutane pyrimidine dimers (CPD) and 6–4 pyrimidine photoproduct. 6–4 PP is only 10–15% of the DNA photolesions caused by UV irradiation, but more lethal [37]. Cisplatin or *cis*-diamminedichloroplatinum (II) (CDDP) is a platinum-based chemotherapeutic agent used to treat a variety of cancers [40], [58] and can cause intrastrand crosslinking of DNA to form abducts Pt-[GG] [40], [45], [58]. IR exposure mainly leads to double stand breaks (DSBs) in DNA, which contribute to the vast majority of human cancers [51]. DNA SSBs can be repaired by the mechanisms of DR, BER, NER and MMR; and DNA DSBs by HR and NHEJ [50], [51]. In mili^-/-^ MEFs, all types of DNA damage except for 6–4 PP were not repaired well, suggesting that Piwil2 is required for the repair of both SSB and DSB. The failed repair was associated with the loss of chromatin decondensation in the mili^-/-^ MEFs. The conclusion is further supported by well-repaired 6–4PP lesions in mili^-/-^ MEFs. Opposed to CPD, which is positioned within nucleosomes, the 6–4PP is formed in the inter-nucleosome linker, exposed on the surface of compact chromatin, and thus accessible to DDR proteins [59]. In addition, the removal of 6–4PP in mili^-/-^ MEFs also suggests that DDR signal transduction pathways down-stream of chromatin relaxation are not impaired in mili^-/-^ MEFs.

The function of Piwil2 on DNA repair may have both positive and negative impacts on tumorigenesis depending on pathophysiological status of a cell. While DNA repair can prevent oncogenetic mutation in normal cells; it might promote tumorigenesis of tumor cells. For example, the majority of traditional anti-cancer drugs are genotoxic, and the resulted DNA-damage may activate *PIWIL*2 gene to promote DNA repair in the targeted tumor cells. As a consequence, the Piwil2-mediated DNA repair may spare the tumor cells from the anti-cancer drug-induced apoptosis. Thus, Piwil2 expression induced by the chemotherapeutic agents such as cisplatin might contribute to drug resistance of tumor cells such as cancer stem cells [60]. This may also explain why little Piwil2 was detected in primary cancers [11].

DNA damage induced by genotoxic agents is the earliest step of tumorigenesis [25], [26], [34]. Normally, the damaged DNA can be repaired correctly through DDR signaling transduction pathways [25], [61]; otherwise erroneous DNA repair may cause activation of oncogene and/or inactivation of tumor suppressor genes, leading to genomic instability, which can in turn promote progression of tumorigenesis [25], [34], [61], [62]. Therefore, the Piwiil2-mediated DNA repair strongly suggests that Piwil2 act as a gatekeeper to genotoxic agents-mediated carcinogenesis and may play a critical role in preventing the initiation and development of a tumor [5], [6], [7], [8], [13], [14]. The mechanism by which DNA damage induces Piwil2 expression is not clear yet. It is likely that cell cycle halting due to DNA damage is required for Piwil2 expression. Further experiments are warranted to elucidate the issue.

Taken together, we have demonstrated that *PIWIL2* can be activated by genotoxic agents to facilitate DNA repair. The Piwil2-mediated DNA repair promotes chromatin relaxation through histone H3 acetylation. Further elucidating how Piwil2 modulates chromatin relaxation may shed new light on the mechanism underlying Piwil2-mediated DNA repair. While DNA damage-associated signaling transduction proteins and DNA repair proteins have been extensively investigated, little is known about the factors that modulate chromatin structure during DDR. The discovery of Piwil2 as a factor for DNA repair opens a novel venue to elucidate the complex network for DNA repair (Figure 6). Therefore, our primary finding that Piwil2 mediates chromatin relaxation to promote DNA repair is of important significance for better understanding of the mechanisms underlying DNA repair, potentially leading to a new concept for tumor development while coupling with other biological functions of Piwil2 [11], [12], [13].

## Materials and Methods

### Animals, antibodies and cell lines

*Piwil2 (mili)* gene knockout mice with C57BL/6 background provided by Dr. Haifan Lin at Department of Cell Biology &Yale Stem Cell Center, Yale University School of Medicine, New Haven, CT, were bred and maintained in the animal pathogen-free facility at The Ohio State University Medical Center. Male C57BL/6 mice were purchased from Jackson Laboratories. The protocol of animal experiments for the study was approved by the Institutional Animal Care and Use Committee (IACUC), OSU (Protocol number: 2006A0250). The following antibodies were used in this study. Rabbit polyclonal antibody to Piwil2 (1∶1000) was generated in our laboratory [11]. Mouse anti-cleaved PARP (1∶1,000), rabbit anti-cleaved caspase-3 (1∶1,000), rabbit anti-Bik (1∶1,000), rabbit anti-Bax (1∶1,000), rabbit anti-Bcl-XL (1∶1,000), rabbit anti-phosphorylated H3 (S10) (1∶1,000) and rabbit anti-AcH3 (K18) (1∶1,000) antibodies were purchased from Cell Signal Technology Inc (Danvers, MA). Rabbit anti-AcH3 (K9,14) (1∶20,000), rabbit anti-AcH3 (K9) (1∶1,000), and rabbit anti-histone H3 (1∶1,000) antibodies were purchased from Millipore (Billerica, MA). Mouse anti-β-Actin (1∶1,000) and mouse anti-Tubulin (1∶2,000) antibodies were purchased from Santa Cruz Biotechnology Inc. (Santa Cruz, CA). Rabbit anti-CPD antibody (1∶1000) was purchased from Sigma. Mouse mAb to anti-64PP (1∶1000) was purchased from MBL International Corporation, Woburn, MA, and rat anti-Pt-GG (1∶1000) was provided by Dr. Jűrgen Thomale, Institut fűr Zellbiologie, Universitätsklinikum Essen, Germany.

Human dermal fibroblasts (HDF)-AI and OSU-2 were used. The HDF-AI were a gift from Dr. Andrew Issekutz, Dalhousie University, Halifax, NS, Canada. Since *PIWIL2* gene can be activated in the stressed culture (unpublished observation), we used subconfluent HDF for experiments. The cell lines were cultured and maintained in D10 F medium (DMEM plus 10% fetal calf serum supplemented with 5 mM glutamine, 50 mM 2-mecaptoethonal, 100 U/ml penicillin, and 100 mg/ml streptomycin).

### Genotyping of mili^-/-^ mice

To obtain mili^-/-^ and wild-type (WT) littermates, male mili^+/-^ mice were crossed with female mii^+/-^ mice. Offsprings were genotyped by genomic DNA Polymerase chain reaction (PCR) [3]. Genomic DNAs of tails were extracted using a silica-gel method with modifications [6], [63], [64] following overnight digestion with 200 µl of DNA lysing buffer (100 mM NaCl, 10 mM Tris-HCl, 25 mM EDTA, 1%SDS, and 50 µg/ml proteinase K) at 56°C. The conditions for genomic DNA PCR were as follows: 10 cycles of initial denaturation at 95°C for 5 min followed by 94°C for 30 s, annealing at 65°C for 1 min, touchdown −1°C/cycle, and extension at 72^o^C for 1 min; and then 25 cycles of 94°C for 30 s, 55°C for 1 min, and 72°C for 1 min with the final step of extension at 72°C for 10 min. All PCR products were separated on 1.0% agarose gel at the 5 v/cm for 90 min. The primer sequences used for PCR were: 5′-ACA TAG CGT TGG CTA CCC GTG ATA-3′ (Neo forward); 5′-TTC ATG CCC ACC TAC CCT GTC CAT -3′ (mili forward); and 5′-GAA AGC TGG CTG TTG TGC CAG TTA-3′ (mili reverse). The expected PCR products were 1250 bp for WT mice and 900 bp mili^-/-^ mice. PCR Master Mix (Promega, Cat No. M7502) was used for all PCR reactions.

### Establishment of mouse embryonic fibroblast (MEF) lines

MEFs were generated from mouse embryos at day 13 post coitum of mili KO and WT mice. Briefly, each embryo was ground in the presence of 1 ml 0.25% trypsin/1 mM EDTA (Gibco, Carlsbad, CA) per embryo, passed through 18 G syringe twice, and incubated at 37°C for 15 min. Trypsin was inactivated by addition of equal volume of DMEM (Gibco) containing 10% fetal bovine serum (FBS; HyClone, South Logan, UT) and the cells of each embryo were then plated in 10 cm culture dishes and allowed to adhere for 24 h. Non-adherent cells were then discarded and the adherent MEFs were expanded by passaging pre-confluent cultures at a ratio 1∶3 or 1∶5. The cell lines were frozen or maintained in D10 F (DMEM plus 10% fetal calf serum supplemented with 5 mM glutamine, 50 mM 2-mecaptoethonal, 100 U/ml penicillin, and 100 mg/ml streptomycin). All cells were cultured at 37°C in a humidified atmosphere of 5% CO_2_. The cultures were split at the log phase of cell growth to prevent over population-induced cell death. The cytology was examined at various time points by Giemsa-staining of cytospin preparations, or directly monitored under a phase contrast microscope.

### Cell survival assay

The sensitivity of mili^-/-^ and WT MEFs to genotoxic agents including IR, UV light, and cisplatin were evaluated by cell survival assay. Cells were seeded into 96-well (3×10^3^/well for mili^-/-^ MEFs and 5×10^5^/well for WT MEFs) for UV light and cisplatin treatment or 6-well plates (1×10^5^/well for both mili^-/-^ and WT MEFs) for X-ray irradiation. The cells were mock treated or treated with various doses of UV light, IR (X-ray), and cisplatin. UV irradiation was performed with a germicidal lamp at a dose rate of 0.8 J/m^2^/s as measured by a Kettering model 65 radiometer (Cole Palmer Instrument Co., Vernon Hill, IL, USA), and X-ray treatment was performed with RS 2000 Biological Irradiator (Rad Source Technologies, Inc. Alpharetta, GA). For cell viability assay of UV light or cisplatin-treated cells, cells were washed in PBS 3 times, fixed in methanol:acidic acid (3∶1) for 1 hr, followed by staining with methylene blue for 1 hr. The plates were then rinsed in cold water, and a 100 µl solution containing 40% methanol, 10% acetic acid was added. Absorbance was measured at 660 nm. For cell survival assay of IR-treated cells, cells were harvested, and counted with trypan blue exclusion of dead cells. The cell survival rate of each sample was normalized to mock- treated counterparts.

### Genomic DNA isolation

Genomic DNA was isolated by using standard techniques described by Sambrook et al [65]. Briefly, cell pellet was lysed in buffer containing 10 mM Tris–HCl (pH 8.0), 0.1 M EDTA, 0.5% SDS during 20 min. Lyzates were incubated with proteinase K (final concentration 100 µg/ml) at 50 °C for 3 h, and extracted twice with phenol and twice with chloroform. Genomic DNA was precipitated with 0.2 volume of ammonium acetate and 2 volumes of ethanol. DNA was washed with 70% ethanol and dissolved in TE buffer. The DNA concentration was determined by spectrophotometry and its integrity was checked by 1.5% agarose gel electrophoresis.

### Immuno-slot blot (ISB) analysis

ISB was used to determine the amounts of CPD, 6–4 PP and Pt-GG. Briefly, DNA (20 µg) isolated from each samples was sonicated and then denatured at 100°C for 10 minutes. The heat-denatured DNA was quickly chilled on ice and immediately slot-blotted onto nitrocellulose membranes using a Convertible Filtration Manifold System (GibcoBRL, Carlsbad, CA). The membranes were baked for 2 hours at 80°C. After the single-stranded DNA was immobilized onto the nitrocellulose membranes, the membranes were blocked with 5% milk−1×TBST and then incubated with antibodies to CPD (1∶1000 diluted), 6–4 PP (1∶1000) Dilution), or Pt-GG (1∶1000 Dlilution) overnight at 4°C. The membrane was then incubated with horseradish peroxidase-conjugated goat anti-mouse or rat IgG (1∶5000 diluted) (Chemicon, Temecula, CA) for 1 hour at 37°C. Chemiluminescent substrate (Super Signal West Dura Extended Duration Substrate, 34075; Pierce Biotech) was used to detect positive bands, which were visualized on X-ray film. The relative amounts of CPD, 6–4 PP and Pt-GG were determined by quantification of the intensity of each band of the lesions and normalization to a reference standards run at the same experiment. The intensity of each band was quantified by scanning images and processing with Alphaimager-2000 software.

### RT-PCR

RT-PCR was performed as previously described [6], [66]. Total RNA was extracted from HDFs and reversely transcribed into cDNA, using Superscriptase II (Invitrogen, CA) and oligo (dT) in a 20 µl reaction containing 1 µg of total RNA, which was pretreated with RNase-free DNase I (Invitrogen, CA) to eliminate contaminating genomic DNA. For PCR, an aliquot of 0.5 µl cDNA was used in each 20 µl PCR reaction, using PCR Master Mix (Promega, MI). The sequences of human Piwil2 primers were as follows: forward 5′-TTCGGAGTGTGGCCCAGAAGATTT -3′ and reverse 5′-ACAGTTCCAGGAGTGGGAGTTACA-3′ with a 499 bp product. The following conditions were used: an initial denaturation at 95°C for 5 min followed by denaturation at 94°C for 30 seconds, annealing at 65°C for 1 min, touchdown −1°C per cycle, and extension at 72°C for 1 min for a total of 10 cycles. Then the condition was fixed for 25 cycles of denaturation at 94°C for 30 seconds, annealing at 50°C for 1 min, and extension at 72°C for 1 min with a final extension at 72°C for 10 min. PCR products were analyzed by 1.5% agarose gel.

### Western Blot

Total cellular proteins were isolated from cultured cells or animal tissues using lysis buffer. Protein concentration was determined by protein assay (D_c_ Protein Assay System; Bio-Rad, Hercules CA), as described by the manufacturer. A total of 40 µg of protein was loaded per well, separated on an SDS-PAGE [8% (w/v) polyacrylamide gel] and then transferred by electrophoresis to nitrocellulose membranes. The membranes were blocked with 5% milk in Tris-buffered saline Tween (M-TBST; 20 mM Tris, 0.5 M NaCl, and 0.05% Tween 20 [pH 7.4]) for approximately 60 minutes at 37°C, incubated overnight at 4°C with a primary antibody appropriately diluted in M-TBST, and rinsed four times in M-TBST. Then, the membranes were incubated with appropriate horseradish peroxidase-conjugated secondary antibody in M-TBST for 1 h at 37°C, rinsed four times with TBST, and developed with chemiluminescent substrate (Super Signal West Dura Extended Duration Substrate, 34075; Pierce Biotech). The positive bands were visualized on X-ray films. Tubulin or β- Actin on the same membrane was used as a loading control.

### Chromatin relaxation assay

Chromatin relaxation was evaluated by MNase digestion [43]. Mili^-/-^ MEFs and WT MEFs were cultured in 6-well plates and irradiated when they became subconfluent. The cells were harvestred immediately after UV irradiation and the nuclei were isolated from mili^-/-^ and WT MEFs, respectively, before and after UV irradiation, which were subjected to MNase digestion as described [43]. The genomic DNA was isolated and the fragments are separated by a 1.8% agarose gel.

### Single-cell gel electrophoresis (Comet assay)

Exponentially growing Mili^-/-^ and WT MEFs cells with 70 – 80% confluence were exposed to radiation at room temperature using a Cabinet X-rays System Faxitron Series (dose rate: 0.997 Gy/min; 130 kVp; Hewlett Packard, McMinnville, OR). Cells sheltered from radiation were included as the sham-IR control. The comet assay was conducted using the Trevigen's CometAssay kit (Alkaline version). Briefly, 1×10^5^/ml cells were mixed with molten LMAgarose (at 37 °C) at a ratio of 1∶10 (v/v) and immediately pietted 50 µl onto the cometSlide and stayed in the dark for 10 min. The slides were then immersed in prechilled lysis solution for 30 min at 4 °C. Excess buffer was drained from slides and the slides were then immersed in freshly prepared alkaline unwinding solution (pH>13) in dark for 30 min at room temperature before electrophoresis at 21 volts for 30 min. The slides were then immersed twice in dH_2_O for 5 min each, then in 70% ethanol for 5 min followed by drying at room temperature for 15 min, staining with DAPI for 5 min and then drying completely at room temperature in the dark. The slides were then viewed by fluorescence microscopy (maximum excitation and emission are respectively 350 nm/470 nm). DNA damage and repair were estimated by measuring the distance of the tail against the edge of far side of the nuclei for 50 random selected cells.

### Detection of γH2AX foci in X-ray treated MEFs

MEFs (Mili^+/+^ and Mili^-/-^) were grown in D10 F medium in an incubator at 37°C with 5% of CO2. The cells were seeded (1×10^6^/ml) on coverslips in a 100 mm culture dish for 2 hrs, grew up to 40% − 60% of confluency prior to X-ray treatment (3 Gy) in triplicate and then were fixed at 1 or 3 hrs after treatment for 10 min in 4% paraformaldehyde. The fixed cells were permeabilized for 5 min at 4°C in 0.5% Triton X-100. The slides were blocked in 1X phosphate-buffered saline (PBS) containing 2% BSA at room temperature for 1 hr. The cells were incubated with mouse monoclonal ***anti*** **-** ***γH2AX*** (phosphor S139) antibody [3F2] (1∶500; Abcam: ab22551) followed by secondary Alexafluor 594 donkey anti-mouse antibody (1∶500; Invitrogen) for 30 min each step at room temperature, and washed three times for each step with 1X PBS. Cell nuclei were counterstained with 4′,6-diamidino-2-phenylindole (DAPI). The slides were analyzed for γH2AX foci under a Nikon E-400 fluorescence microscope.

### Statistical analysis

Data of multiple group observations were statistically analyzed by the one-way analysis of variance (ANOVA), and two groups of observations were compared by student-T test. A value of p≤0.05 was considered significant. Data are expressed as mean±SD. *, p≤0.05; **, p≤0.01.
