# Correction: Germline Stem Cell Gene *PIWIL2* Mediates DNA Repair through Relaxation of Chromatin

**DOI:** 10.1371/annotation/2c1c26ac-ee7e-417d-9852-4d2995ad102b

**Published:** 2011-11-30

**Authors:** De-Tao Yin, Qien Wang, Li Chen, Meng-Yao Liu, Chunhua Han, Qingtao Yan, Rulong Shen, Gang He, Wenrui Duan, Jian-Jian Li, Altaf Wani, Jian-Xin Gao

There was an error in the funding statement. The correct funding statement is: "This work is supported by the start-up funding from Renji Hospital Clinical Stem Cell Center, Shanghai Jiao Tong University School of Medicine and Med-X institute, China (JXG and MYL); National Natural Science Foundation of China (NSFC 81171940; JXG and MYL); Strategy Initiative Grant (SIG) 2006/2007 (OSU Medical College; JXG); Immunology Program Award 2008 (OSUCCC; JXG); American Cancer Society IRG-112367 (JXG); Davis/Bremer Medical Research Grant (OSU; RH and JXG); Department Start-up funding (GH); Chinese scholarship (China Scholarship Council (DTY); and Grant CA93413 (NCI; AW). The funders had no role in study design, data collection and analysis, decision to publish, or preparation of the manuscript." 

